# Supplementary material for: Psychometric properties of the brief self-report questionnaire for screening putative pre-psychotic states and validation of clinical utility in young adult
Source: PLoS One. 2021 Jun 17;16(6):e0251915. doi: 10.1371/journal.pone.0251915 (PMC8211275; doi:10.1371/journal.pone.0251915)
Supplement: Attachment — Submitted filename: Response t Reviewers.docx [file pone.0251915.s002.docx]

To academic editor

1. Please ensure that your manuscript meets PLOS ONE's style requirements, including those for file naming. The PLOS ONE style templates can be found at <https://journals.plos.org/plosone/s/file?id=wjVg/PLOSOne_formatting_sample_main_body.pdf> and https://journals.plos.org/plosone/s/file?id=ba62/PLOSOne_formatting_sample_title_authors_affiliations.pdf

Answer: We had checked and confirmed that our manuscript meets PLOS ONE’s style requirements.

2. We note that Table 1 may include questionnaire items that may have been previously published. The reproduction of previously published work has implications for the copyright that may apply to these publications. We would be grateful if you could clarify whether you have obtained permission from the original copyright holder to republish these items under a CC BY license. If you have not obtained permission to publish these items please remove them from your manuscript. You may wish to replace the text you have removed with relevant question numbers/ brief descriptions of each item; please be sure to include any relevant references and in-text citations.

Answer: The third author of this manuscript is the original copyright holder of the BQSPS. Without any doubt, we got his permission before this study began. We also clarify this in the manuscript.

3. We note that you have stated that you will provide repository information for your data at acceptance. Should your manuscript be accepted for publication, we will hold it until you provide the relevant accession numbers or DOIs necessary to access your data. If you wish to make changes to your Data Availability statement, please describe these changes in your cover letter and we will update your Data Availability statement to reflect the information you provide.

Answer: We do not change our Data Availability statement. However, we changed our data sharing method from providing a DOI number into providing Supporting information files in this study.

4. Please review your reference list to ensure that it is complete and correct. If you have cited papers that have been retracted, please include the rationale for doing so in the manuscript text, or remove these references and replace them with relevant current references. Any changes to the reference list should be mentioned in the rebuttal letter that accompanies your revised manuscript. If you need to cite a retracted article, indicate the article’s retracted status in the References list and also include a citation and full reference for the retraction notice.

Answer: We did not cite any articles that have been retracted. For answering the third point of additional editor comments, we add a new citation, “Tabachnick BG, Fidell LS. Using multivariate statistics (5^th^ ed.). Boston, MA: Allyn & Bacon; 2007”.

Additional Editor Comments:

1) Please, use "construct validity" instead of "constructive validity".
2) Please, use "components" instead of "factors" as you run PCA not EFA.
3) Please, do not say that factor loadings were significant, as you did not report their p values. Usually, we retain an item based on a pre-established cut-point (e.g. >.30 or >. 40) not based on statistical significance.
4) In the Discussion, a mention of your study limitations is missing; therefore, please, add this part.

Answer: We have revised these minor issues in the manuscript accordingly.

To reviewer 1

1. Is the manuscript technically sound, and do the data support the conclusions?

   The manuscript must describe a technically sound piece of scientific research with data that supports the conclusions. Experiments must have been conducted rigorously, with appropriate controls, replication, and sample sizes. The conclusions must be drawn appropriately based on the data presented.

Reviewer #1:Yes

Answer: Thank the reviewer's comment.

2. Has the statistical analysis been performed appropriately and rigorously?

Reviewer #1:Yes

Answer: Thank the reviewer's comment.

3. Have the authors made all data underlying the findings in their manuscript fully available?

   The [PLOS Data policy](http://www.plosone.org/static/policies.action#sharing) requires authors to make all data underlying the findings described in their manuscript fully available without restriction, with rare exception (please refer to the Data Availability Statement in the manuscript PDF file). The data should be provided as part of the manuscript or its supporting information, or deposited to a public repository. For example, in addition to summary statistics, the data points behind means, medians and variance measures should be available. If there are restrictions on publicly sharing data—e.g. participant privacy or use of data from a third party—those must be specified.

Reviewer #1:Yes

Answer: Thank the reviewer's comment.

4. Is the manuscript presented in an intelligible fashion and written in standard English?

   PLOS ONE does not copyedit accepted manuscripts, so the language in submitted articles must be clear, correct, and unambiguous. Any typographical or grammatical errors should be corrected at revision, so please note any specific errors here.

Reviewer #1:Yes

Answer: Thank the reviewer's comment.

5. Review Comments to the Author
Reviewer #1: Identification of psychosis in the early stage is very much fruitful regarding the clinical outcome. So, this study is a good effort to meet the burning issue.
The study was found well organized, used various appropriate statistical analysis tools, and was well written.
I would like to provide some minor suggestions-
1)The full form of ROC should be mentioned at the beginning
2)Psychometric properties of all Chinese versions should be specifically mentioned (Cronbach’s alpha etc.)
3)The statistical part seemed perfect from my point of view, but require further scrutiny by other experts.

Answer: Thank the reviewer's comment. For suggestions #1 and #2, we have revised these minor issues in the manuscript accordingly. We also expected to get a critical suggestion about suggestion 3.

6. PLOS authors have the option to publish the peer review history of their article ([what does this mean?](https://journals.plos.org/plosone/s/editorial-and-peer-review-process#loc-peer-review-history)). If published, this will include your full peer review and any attached files.

If you choose “no”, your identity will remain anonymous but your review may still be made public.

**Do you want your identity to be public for this peer review?** For information about this choice, including consent withdrawal, please see our [Privacy Policy](https://www.plos.org/privacy-policy).

Reviewer #1:**Yes:** Panchanan Acharjee

Answer: Thank the reviewer's comment.

To reviewer 2

1. Is the manuscript technically sound, and do the data support the conclusions?

   The manuscript must describe a technically sound piece of scientific research with data that supports the conclusions. Experiments must have been conducted rigorously, with appropriate controls, replication, and sample sizes. The conclusions must be drawn appropriately based on the data presented.

Reviewer #2:Yes

Answer: Thank the reviewer's comment.

2. Has the statistical analysis been performed appropriately and rigorously?

Reviewer #2:Yes

Answer: Thank the reviewer's comment.

3. Have the authors made all data underlying the findings in their manuscript fully available?

   The [PLOS Data policy](http://www.plosone.org/static/policies.action#sharing) requires authors to make all data underlying the findings described in their manuscript fully available without restriction, with rare exception (please refer to the Data Availability Statement in the manuscript PDF file). The data should be provided as part of the manuscript or its supporting information, or deposited to a public repository. For example, in addition to summary statistics, the data points behind means, medians and variance measures should be available. If there are restrictions on publicly sharing data—e.g. participant privacy or use of data from a third party—those must be specified.

Reviewer #2:Yes

Answer: Thank the reviewer's comment.

4. Is the manuscript presented in an intelligible fashion and written in standard English?

   PLOS ONE does not copyedit accepted manuscripts, so the language in submitted articles must be clear, correct, and unambiguous. Any typographical or grammatical errors should be corrected at revision, so please note any specific errors here.

Reviewer #2:No

Answer: Thank the reviewer's comment. An experienced academic native English speaker edited the paper.

5. Review Comments to the Author

Reviewer #2: I understand that this study was performed to validate the psychometric properties of the BQSPS by comparing the results with 2 other previous studies which had the same objective yet a distinctively different population..
The main objective of the study was feasible and interesting yet in the execution I found myself to be lost ..
As the author compared some other studies which used different scales and although the comparisons might be richer this way ..the main objective is lost in the way and the discussion section became overcrowded and confusing.. focusing on the author’s main objective can make the reader more interested in the results.
Table 1 is not well organized, some points are affirmatives others are questions.
Table 2& 3 are better to follow the same items sequence not to have the reader confused .

Finally, the manuscript should be reviewed by a native English speaker for the quality of language.

Answer: Thank the reviewer's comment. We had added some sentences to make the paragraph more clear for the readers. Besides, an experienced academic native English speaker edited the paper.

6. PLOS authors have the option to publish the peer review history of their article ([what does this mean?](https://journals.plos.org/plosone/s/editorial-and-peer-review-process#loc-peer-review-history)). If published, this will include your full peer review and any attached files.

If you choose “no”, your identity will remain anonymous but your review may still be made public.

**Do you want your identity to be public for this peer review?** For information about this choice, including consent withdrawal, please see our [Privacy Policy](https://www.plos.org/privacy-policy).

Reviewer #2:**No**

Answer: Thank the reviewer's comment.
